# Predicting Pseudogene–miRNA Associations Based on Feature Fusion and Graph Auto-Encoder

**DOI:** 10.3389/fgene.2021.781277

**Published:** 2021-12-13

**Authors:** Shijia Zhou, Weicheng Sun, Ping Zhang, Li Li

**Affiliations:** ^1^ Hubei Key Laboratory of Agricultural Bioinformatics, College of Informatics, Huazhong Agricultural University, Wuhan, China; ^2^ Hubei Hongshan Laboratory, Huazhong Agricultural University, Wuhan, China

**Keywords:** pseudogene, microRNA, ceRNA network, feature fusion, graph auto-encoder, extreme gradient boosting

## Abstract

Pseudogenes were originally regarded as non-functional components scattered in the genome during evolution. Recent studies have shown that pseudogenes can be transcribed into long non-coding RNA and play a key role at multiple functional levels in different physiological and pathological processes. microRNAs (miRNAs) are a type of non-coding RNA, which plays important regulatory roles in cells. Numerous studies have shown that pseudogenes and miRNAs have interactions and form a ceRNA network with mRNA to regulate biological processes and involve diseases. Exploring the associations of pseudogenes and miRNAs will facilitate the clinical diagnosis of some diseases. Here, we propose a prediction model PMGAE (Pseudogene–MiRNA association prediction based on the Graph Auto-Encoder), which incorporates feature fusion, graph auto-encoder (GAE), and eXtreme Gradient Boosting (XGBoost). First, we calculated three types of similarities including Jaccard similarity, cosine similarity, and Pearson similarity between nodes based on the biological characteristics of pseudogenes and miRNAs. Subsequently, we fused the above similarities to construct a similarity profile as the initial representation features for nodes. Then, we aggregated the similarity profiles and associations of nodes to obtain the low-dimensional representation vector of nodes through a GAE. In the last step, we fed these representation vectors into an XGBoost classifier to predict new pseudogene–miRNA associations (PMAs). The results of five-fold cross validation show that PMGAE achieves a mean AUC of 0.8634 and mean AUPR of 0.8966. Case studies further substantiated the reliability of PMGAE for mining PMAs and the study of endogenous RNA networks in relation to diseases.

## Introduction

In mammalian genomes, only about 1–2% of genes encode proteins (Carninci et al., 2005). The remaining parts involve non-coding RNAs, including pseudogenes, long non-coding RNAs (lncRNAs), and miRNAs. Pseudogenes usually refer to DNA sequences similar to genes but lack coding function in the genome. However, there is increasing evidence showing that pseudogenes can be transcribed into non-coding RNAs and become important regulators in organisms, especially in human cancer (Ma et al., 2021). Some of them may be potential therapeutic targets (Shi et al., 2015). The study of pseudogenes may help the diagnosis or clinical treatment of cancer. miRNAs are short non-coding RNAs between 19 and 25 nucleotides in length, accounting for about 3% of the genome (Setoyama et al., 2011). miRNAs regulate gene expression by acting on mRNAs to affect many developmental processes and the occurrence of diseases (Plank, 2014; Santulli, 2015; Liu Z. et al., 2016). On the other hand, miRNAs can be used as biomarkers for the objective evaluation and diagnosis of tumors (Ruan et al., 2009; Zhang et al., 2012; Stiegelbauer et al., 2014).

Pseudogenes and miRNAs are important components of the competing endogenous RNA (ceRNA) network (Karreth et al., 2015). ceRNAs can regulate gene expression by competing with miRNAs to construct a ceRNA network (Salmena et al., 2011; Rutnam et al., 2014). The ceRNA network can be understood as a balancing mechanism regulating cell activities at the RNA level. Exploring molecular associations in the ceRNA network helps in finding more biological mechanisms at the RNA level. It is important to study various associations in the ceRNA network but this process is often time-consuming and it can be laborious to study the associations by wet experiments. Various computational methods have been developed accordingly.

Currently, non-coding RNA associations in the ceRNA network have been predicted by diverse machine learning methods, which mainly fall into three categories. The first category is based on matrix factorization (MF). MF extracts features by decomposing the input matrix into the product of two or more low-rank matrices. For instance, Zhang et al. proposed a graph-regularized generalized matrix factorization model for predicting a variety of biomolecular interactions (Zhang et al., 2020). Chen et al. and Xu et al. predicted the miRNA–disease associations based on the probability matrix decomposition and inductive matrix completion, respectively (Chen et al., 2018; Xu et al., 2019). Zheng et al. and Liu et al. respectively introduced methods based on collaborative matrix factorization and neighborhood-regularized logistic matrix factorization to predict drug–target interactions (Zheng et al., 2013; Liu Y. et al., 2016). The second category is based on graph embedding. The known associations are learned by the graph embedding method to obtain the behavior information of nodes, and then the characteristics are fused with the characteristic information of nodes, and then the classifiers use node features to predict results. Ji et al. predicted miRNA–disease associations based on the GraRep embedding model (Ji et al., 2020). Song et al. predicted lncRNA–disease associations based on the DeepWalk embedding model (Song et al., 2020). The third category is based on deep learning, among which the most representative method is the graph convolution network (GCN). The GCN is an end-to-end learning model that can deeply integrate the feature information and topological relationship of nodes in the network. Fu et al. proposed a deep learning model based on the multi-view GCN to predict multiple molecular associations (Fu et al., 2021). Xuan et al. and Long et al. proposed GCNLDA and GCNMDA based on the GCN to predict lncRNA–disease associations and microbe-drug associations, respectively (Xuan et al., 2019; Long et al., 2020).

Although pseudogenes play an important role in the ceRNA network, the computational study of associations between pseudogenes and miRNAs is under-developed. Here, we presented a method predicting pseudogene–miRNA associations (PMAs) based on feature fusion and GAE. Given there are many prediction models that can accurately predict lncRNA–miRNA associations, we proposed that the role of pseudogenes is comparable to that of lncRNAs in the ceRNA network. Thus, the expression level can be used as the node feature for pseudogenes as the methods focus on lncRNAs. We fused the node features into the pseudogene–miRNA network and predicted PMAs by a computational method. To the best of our knowledge, this is the first attempt at PMA prediction. The model achieves the mean area under the ROC curve (AUC) and mean area under the precision–recall curve (AUPR) of 0.8634 and 0.8966, respectively. The experimental results confirmed PMGAE-predicted potential PMAs. We also demonstrated the performance of PMGAE through a series of comparative experiments. Together, PMGAE is a powerful and reliable method for the prediction of PMAs as an important component of the ceRNA network.

## Materials and Equipment

### Datasets

We downloaded known PMAs from starBase v2.0 (Li et al., 2014), a large miRNA database that includes the association between miRNAs and lncRNAs and their associations with mRNAs, pseudogenes, and proteins. dreamBase (Zheng et al., 2018) is a database containing massive pseudogene information, including the associations between pseudogenes and the transcription factor (TF), the connection with RNA-binding protein (RBP), and the expression level of pseudogenes in various normal tissues or cancer tissues. We obtained the expression level of pseudogenes in various tissues as the characteristic information of pseudogenes. miRBase (Kozomara et al., 2019) is a comprehensive miRNA sequence database, which contains miRNA sequence information. We obtained the miRNA sequence as the characteristic information of miRNAs from it.

### Data Preprocessing

After quality checking and filtering the obtained data, the dataset comprises the expression information of 444 pseudogenes, the sequence information of 173 miRNAs, and 1,884 pairs of pseudogene–miRNA associations. In addition, considering the independence of the testing set used in the case study, we firstly divided all association pairs into two parts. One is used for model training, and the other is used for the case study.

miRNA sequences are composed of four types of nucleotides: A, adenine; G, guanine; C, cytosine; U, uracil. We set k in k-mer to 3, and each miRNA sequence can be represented as a 64 (4 × 4 × 4)-dimensional vector, where each dimension can represent the frequency of each 3-mer sequence in the sequence. For example, in the miRNA sequence “AGGUUCCAGG,” p (“AGG”) = 2/(10−3+1). For the pseudogenes, we normalized the expression level of pseudogenes as their characteristics.

For the PMAs, we construct a 444 × 173 PMA matrix and put the known PMAs into the PMA matrix. If the ith pseudogene is associated with the jth  miRNA, then let PMA(i,j) = 1; otherwise, let PMA(i,j) = 0.

## Methods

### PMGAE Overview

**FIGURE 1:**
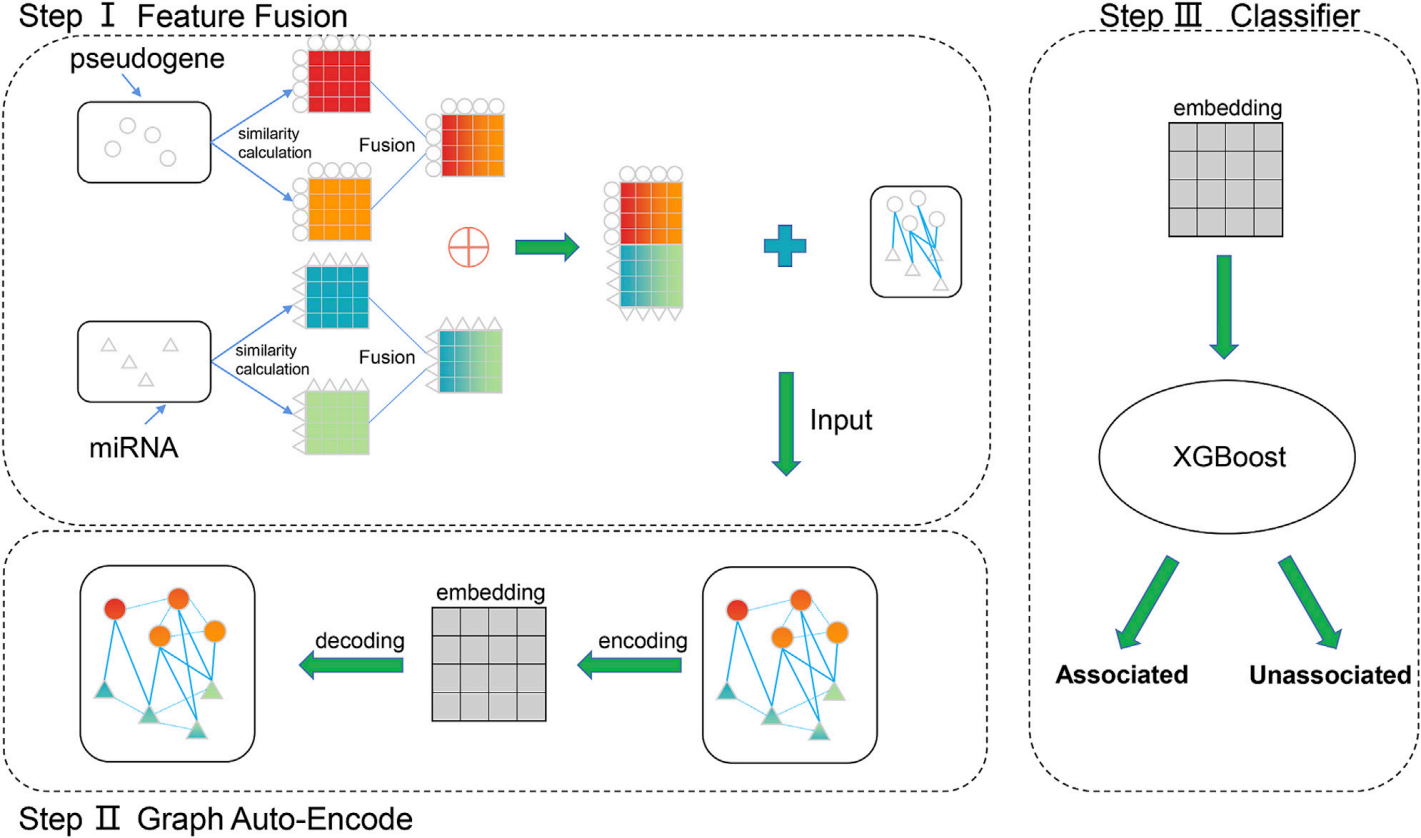
Flowchart of PMGAE.

PMGAE is composed of three steps, as shown in Figure 1. In step Ⅰ, we calculated and fused the biological characteristics of pseudogenes and miRNAs to obtain the similarity profiles as their features. In step Ⅱ, we obtained the low-dimensional representation vector of nodes by a GAE based on the feature information and association information of existing nodes. In step Ⅲ, we fed the low-dimensional vector into XGBoost to predict the PMAs.

### Feature Fusion

We computed the Jaccard similarity coefficient, cosine similarity coefficient, and Pearson similarity coefficient based on the respective characteristics of pseudogenes and miRNAs. We calculated Gaussian kernel similarity based on PMAs to replace the zeros in the matrix (Chen, 2015). Eventually, we generated the pseudogene similarity (PS) profile of 444 × 444 in dimension and the miRNA similarity (MS) profile of 173 × 173 in dimension. Jaccard similarity, cosine similarity, and Pearson similarity can be calculated as follows:

$$\begin{array}{l} Jaccard\left(X,Y\right)=\frac{X\cap Y}{X\cup Y},\\ \text{C}os\left(x,y\right)=\frac{\sum_{k=1}^{n}x_{k}y_{k}}{\sqrt{\sum_{k=1}^{n}x_{k}^{2}}\sqrt{\sum_{k=1}^{n}y_{k}^{2}}},\\ \rho_{X,Y}=\frac{cov\left(X,Y\right)}{\sigma_{X}\sigma_{Y}}=\frac{E\left(XY\right)-E\left(X\right)E\left(Y\right)}{\sqrt{E\left(X^{2}\right)-E^{2}\left(X\right)}\sqrt{E\left(Y^{2}\right)-E^{2}\left(Y\right)}}. \end{array}$$ (1)

Individual similarity measures between pseudogenes and between miRNAs may contain noise in the data. In order to reduce the noise, we fused several similarity profiles by feature fusion. Feature fusion obtains a single output matrix by fusing all similarity profiles with non-linear methods (Wang et al., 2014). Firstly, we construct the weight matrix as

$$P\left(i,j\right)=\left\{ \begin{array}{r} \frac{S\left(i,j\right)}{2\sum_{k\neq i}S\left(i,k\right)},i\neq j\\ 1/2,i=j \end{array}\right..$$ (2)

The local affinity matrix is defined as

$$L\left(i,j\right)=\left\{ \begin{array}{r} \frac{S\left(i,j\right)}{\sum_{k\in N_{i}}S\left(i,k\right)},j\in N_{i}\\ 0,otherwise \end{array}\right.,$$ (3)

where S(i,j) represents the similarity matrix and $N_{i}$ represents neighbors of the ith node. Then, we iteratively update the matrix as

$$P_{t+1}^{\left(v\right)}=L^{\left(v\right)}\times \left(\frac{\sum_{k\neq v}P_{t}^{k}}{n-1}\right)\times {\left(L^{\left(v\right)}\right)}^{T},v=1,2,...,n.$$ (4)

The final feature matrix (here, we set *n* to 3 in our model) is represented as

$$P_{t}=\frac{p_{t}^{\left(1\right)}+p_{t}^{\left(2\right)}+...+p_{t}^{\left(\text{n}\right)}}{n}.$$ (5)

For the fusion similarity profiles PS and MS , we removed the noise by a stacked auto-encoder (SAE) and obtained the low-dimensional vector representation of pseudogenes and miRNAs. By an SAE, we obtained 128-dimensional matrix representations of $PS^{\text{'}}$ and $MS^{\text{'}}$ for pseudogenes and miRNAs, respectively. Finally, in order to improve the training speed and prediction effect of the model, we tried to standardize the obtained 128-dimensional vectors. Specifically, we carried it out using StandardScaler and RobustScaler individually. StandardScaler and RobustScaler can be expressed as

$$\begin{array}{l} x^{\prime }=\frac{x-\mu }{\sigma },\\ y^{\prime }=\frac{y-median}{IQR}, \end{array}$$ (6)

where IQR represents the interquartile range of the sample.

StandardScaler improves the rate of learning and prediction accuracy of the model. RobustScaler reduces the effect of outliers on results. Both of them are important, so we took the mean values of the matrix that are treated by each of them separately and obtained the final feature matrices $PS^{\prime \prime }$ and $MS^{\prime \prime }$ . Finally, the node feature matrix X is constructed as

$$X=\left(\begin{array}{c} PS^{\prime \prime }\\ MS^{\prime \prime } \end{array}\right).$$ (7)

### Graph Auto-Encoder

Auto-encoder is a kind of neural network, which can restore the input using output through certain training. It includes an encoder and a decoder. The encoder obtains the low-dimensional representation of the input vector (Baldi, 2012). The GAE migrates the auto-encoder to a graph (Kipf and Welling, 2016). We constructed the adjacency matrix and the feature matrix of the nodes. The goal is to obtain the low-dimensional representation of the nodes by deeply integrating the association information between nodes and the feature information of nodes themselves through the GAE. The GAE uses a two-layer graph convolution network as an encoder, which can be described as follows:

GCN(X,A)=A∼ReLu(A∼XW0)W1, (8)

where $\tilde{A}=D^{-\frac{1}{2}}AD^{-\frac{1}{2}}$ , ReLu(X)=max(X,0) represents the activation function, and $W_{0}$ and $W_{1}$ are parameters to be learned.

We built the adjacency matrix based on the PMA network as follows:

$$A=\left(\begin{array}{cc} 0 & PMA\\ PMA^{T} & 0 \end{array}\right),$$ (9)

where $PMA^{T}$ represents the transpose of the matrix PMA .

We used the adjacency matrix A and feature matrix X to obtain the low-dimensional representation vector of nodes by an encoder, which can be defined as

Z=GCN(X,A). (10)

The decoder also obtains the low-dimensional vector recomposition map based on the neural network. The decoder generates a graph according to the probability of edges between nodes. It can be defined as

$$\hat{A}=sigmoid\left(ZZ^{T}\right),$$ (11)

where $sigmoid\left(x\right)=\frac{1}{1+e^{-x}}$ represents the activation function. $\hat{A}$ is the reconstructed network matrix. In this study, in order to make the model more explanatory, we do not use the decoder layer but put the low-dimensional representation vector of nodes into the best classifier we trained to predict the PMAs.

To measure the error between the predicted and the real association, the loss function is defined as

$$L=-\frac{1}{N}\sum y\log\hat{y}+\left(1-y\right)\log\left(1-\hat{y}\right),$$ (12)

where y represents the value of an element in the adjacency matrix A (0 or 1) and $\hat{y}$ represents the value of the same element in the reconstructed adjacency matrix $\hat{A}$ (0–1). We took multiple epochs to minimize the loss function to make the reconstituted data as similar to the original data as possible.

Subsequently, we predicted potential PMAs by XGBoost. XGBoost is a machine learning algorithm whose core idea is to integrate multiple decision trees and continuously add trees to them. Each addition of trees is a process of iteratively adding new functions. Its purpose is to make the final predicted value as close as possible to the real value. Its implementation process can be expressed as

$${\hat{y_{i}}}^{\left(t\right)}=\sum_{k=1}^{t}f_{k}\left(x_{i}\right)={\hat{y_{i}}}^{\left(t-1\right)}+f_{t}\left(x_{i}\right).$$ (13)

The objective function of XGBoost is defined as follows:

$$L\left(\varphi \right)=\sum_{i}l\left(y_{i},{\hat{y}}_{i}\right)+\sum_{k}\Omega \left(f_{k}\right),$$ (14)

where $l\left(y_{i},\hat{y_{i}}\right)$ is the training error and $\Omega \left(f_{k}\right)$ is the regularization term to suppress over-fitting.

### Graph Embedding

In contrast to the traditional machine learning algorithm which may only consider the mapping from input to output without considering the associations in the network, the graph-based algorithm can obtain the associations between nodes together with their own characteristics to improve the accuracy of prediction. The graph data we obtain from real life are often high-dimensional and sparse. Graph embedding is the process of mapping the input graph data to low-dimensional dense vectors, which can reinforce the efficiency of machine learning and improve the accuracy of prediction.

We selected several representative graph embedding methods including Line (Tang et al., 2015), GraRep (Cao et al., 2015), Node2vec (Grover and Leskovec, 2016), and DeepWalk (Perozzi et al., 2014) to predict the PMAs and compared the results of PMGAE in *Results*.

## Results

### Experimental Setup and Performance Evaluation

For the experiment parameters in the GAE, we set a learning rate of 0.001 and trained the model for 8,000 epochs. We obtained a 32-dimensional representation for each node. Then, they were put into XGBoost for prediction. In addition, we used five-fold cross validation to evaluate the performance of the model. We take the known PMAs as a positive sample. The remaining unknown PMAs can be considered potential negatives from which we randomly selected PMAs with equal size to the positive samples as negative samples. Subsequently, we randomly divided the positive and negative samples into five parts. One in the five parts was taken out in turn as a test set, and the remaining were used as the training sets.

**FIGURE 2:**
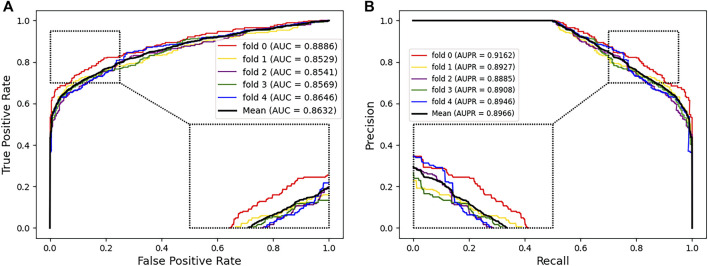
AUC **(A)** and AUPR **(B)** of PMGAE using five-fold cross validation. Insets represent the zoom-in view of local regions.

We used several evaluation metrics including accuracy, sensitivity, specificity, and precision. In addition, we also adopted the AUC and AUPR to evaluate the prediction performance. We took multiple independent experiments of five-fold cross validation to reduce the error. The mean AUC and AUPR were shown under the corresponding curve (Figure 2). The AUC and AUPR of our prediction model reached 0.8634 and 0.8966, respectively, which showed that PMGAE has satisfactory performance in PMA prediction.

### Comparison of the Performance of PMGAE and MF-Based Methods

MF-based methods have shown excellent performance in predicting the correlation of various biomolecules. To evaluate the performance of PMGAE, we compared it with MF-based methods including multiple similarities collaborative matrix factorization (MSCMF), inductive matrix completion for miRNA–disease association (IMCMDA), and neighborhood-regularized logistic matrix factorization (NRLMF). MSCMF is a collaborative filtering model integrating multiple similarities for predicting drug–target interactions (Zheng et al., 2013). IMCMDA is a matrix completion–based model, integrating miRNA–disease associations, individual miRNA and disease characteristics, and Gaussian interaction profile kernel similarity between them to predict miRNA–disease associations (Chen et al., 2018). NRLMF combined logical matrix factorization and neighborhood regularization to predict drug–target interactions (Liu Y. et al., 2016).

**FIGURE 3:**
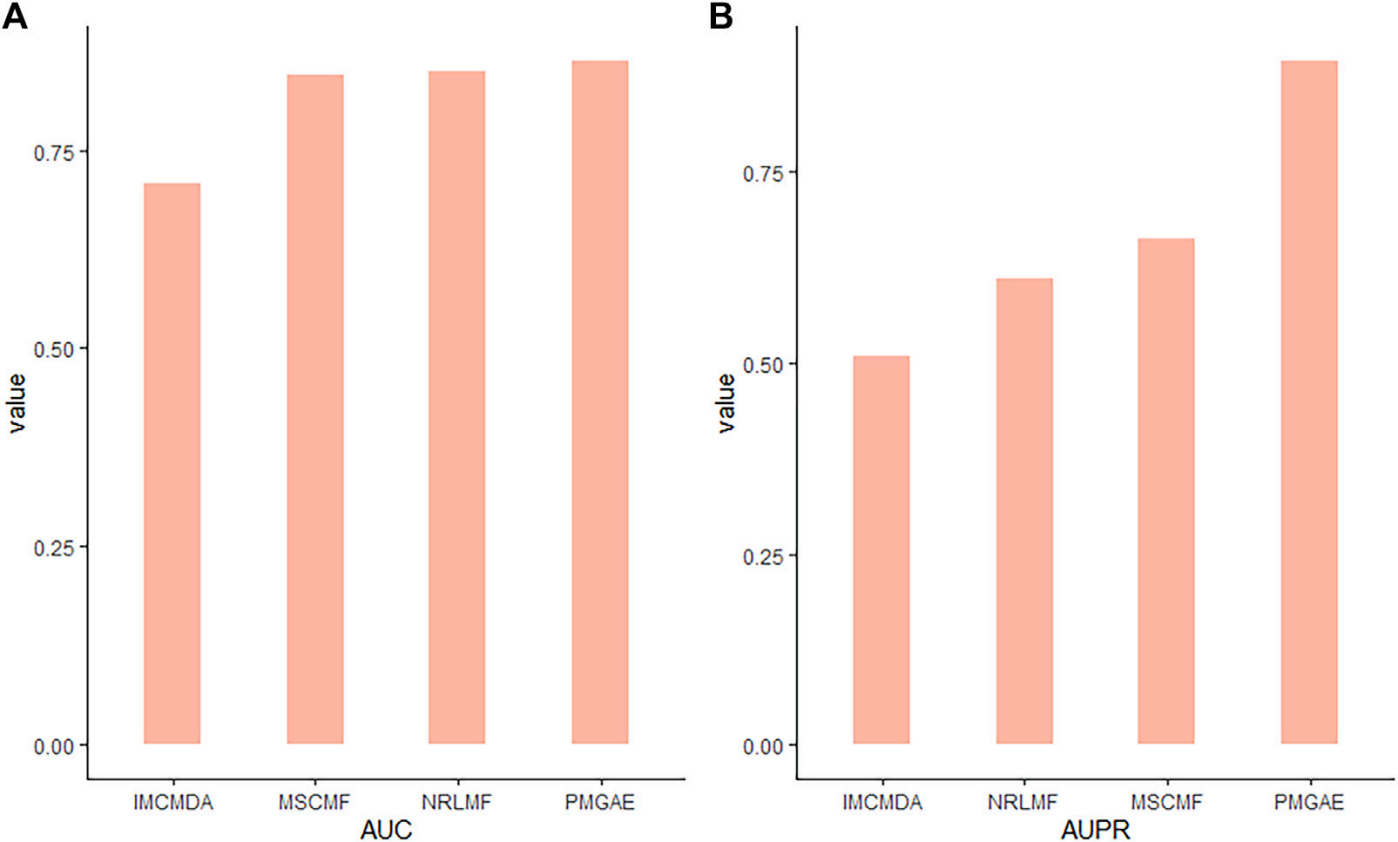
Comparison of AUC **(A)** and AUPR **(B)** of PMGAE and MF-based models.

As shown in Figure 3, PMGAE showed the best performance in terms of AUC and AUPR. Relative to the MF-based methods, the GAE can effectively extract node features, with the best prediction achieved through XGBoost.

### Visualization of Embedding Effect

Because the features are high-dimensional, it is difficult to visualize the clustering results directly. In order to make the model more interpretable and validate the embedded effects, we mapped the features of the nodes before and after embedding them into the three-dimensional space through t-SNE (Maaten and Hinton, 2008). t-SNE can reduce the high-dimensional data to two or three dimensions. Through t-SNE, we can do an intuitive observation on the embedding method for the node clustering effect.

**FIGURE 4:**
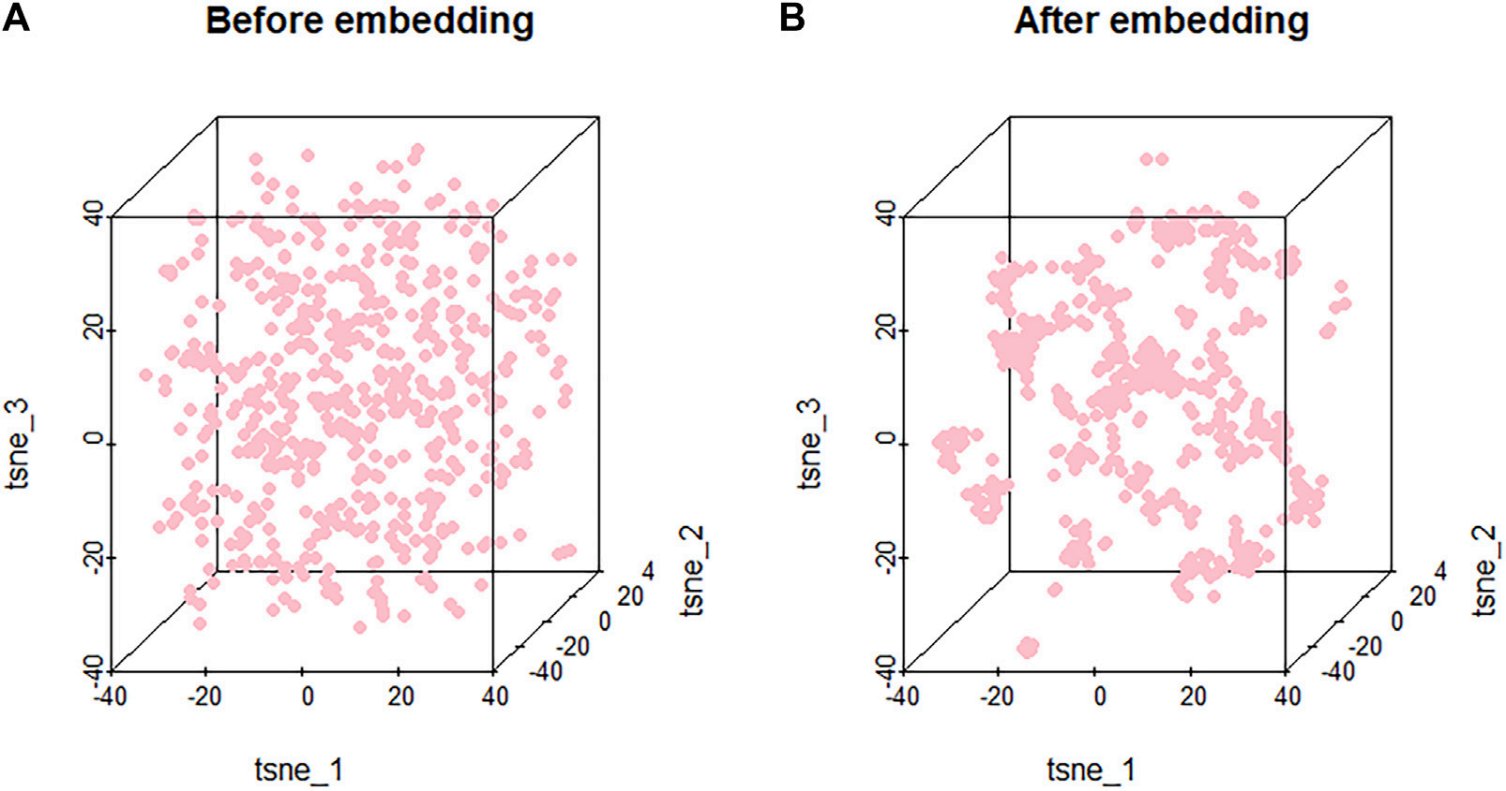
Clustering results of nodes before **(A)** and after **(B)** embedding.

As shown in Figure 4, nodes are randomly distributed before embedding, and our embedding method leads to clustering of the nodes based on their characteristics. Since similar molecules may have similar or related biological functions, effective clustering can facilitate potential association prediction and improve the performance of the model. The effective clustering through embedding validates it as an important component of PMGAE.

### Feature Fusion With Various Similarity Measures

Using the expression information of pseudogenes and the k-mer sequence information of miRNAs, we calculated the Jaccard similarity coefficient, cosine similarity coefficient, and Pearson similarity coefficient of pseudogenes and miRNAs, respectively. Then, pairwise fusion and full fusion were performed and compared. Table 1 shows the performance of specific fusions and no fusion.

**TABLE 1:** Model performance comparison using similarity profile fusions and using individual similarity profiles.

Methods	Evaluation metrics					
	Acc.	Sen.	Spec.	Prec.	AUC	AUPR
Jaccard	0.7641	0.6443	0.8838	0.8475	0.8416	0.8676
Pearson	0.7633	0.6555	0.8710	0.8356	0.8381	0.8637
Cosine	0.7901	0.6491	0.9310	0.9040	0.8562	0.8872
Cosine + Jaccard	0.7927	0.6433	0.9421	0.9176	0.8607	0.8912
Cosine + Pearson	0.7964	0.6396	0.9533	0.9320	0.8591	0.8935
Jaccard + Pearson	0.7954	0.6460	0.9448	0.9214	0.8565	0.8913
Full fusion	0.8015	0.6592	0.9437	0.9216	0.8632	0.8966

Individual similarity has its own limitations. For example, the cosine similarity coefficient tends to distinguish differences from directions; thus, it has a good effect on the calculation of different directions but is not sensitive to the change of values. The Jaccard similarity coefficient has a good effect on the binary data, but it cannot measure the specific value of the difference. The Pearson similarity coefficient tends to give better results when the data do not conform to a certain rule, but the effect on overlapping data is compromised. Considering these shortcomings, we tried to fuse these similarity measures in a non-linear way for a better similarity representation by integrating the advantages. The experimental results in Table 1 show that our full similarity fusion method can effectively improve the performance of the model.

### Comparison of the Performance of Various Embedding Methods

**FIGURE 5:**
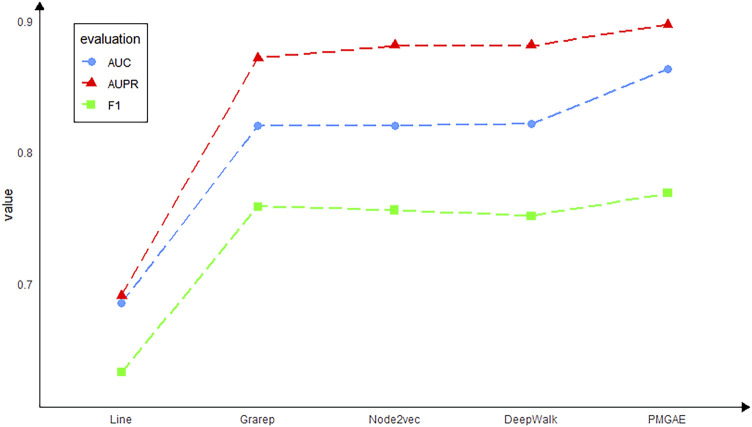
Model performance using various embedding methods.

For each method, the mean of individual runs is used to measure its performance. As shown in Figure 5, the PMGAE model shows the best prediction. The performance of GAE is superior to that of other graph embedding methods. The GAE more effectively mines the topology structure in the scenario of node information in the network than other embeddings.

Although the graph embedding models mentioned above have many advantages, according to our experimental study, we found that these models still have some drawbacks. Specifically, the Line model only considers the first-order relationship and second-order relationship of nodes. It cannot construct the global structure of the network well, and the embedding of Line for low-level nodes is not accurate enough. Thus, the prediction outcome of Line is the least accurate in our data. DeepWalk takes into account each first-order relationship of the node with all relationships stored in a subspace. But it cannot distinguish the order of the node’s neighbors during training. At the same time, DeepWalk is only applicable to unweighted graphs and has obvious limitations. The Node2vec model combines some advantages of Line and DeepWalk and also can control the preference of random walk by adjusting the hyperparameters. However, when the number of samples is limited as in the case of PMGAE, the length of random walk is also limited. So, the learning effect for remote neighbors in the network is far from optimum. The GraRep model can put each first-order relationship between nodes in different subspaces, which well constructs the global structure of the network. However, the calculation of each first-order relationship $A^{k}$ and the optimization loss function is large, so it cannot be used for large-scale graph data. Besides, the above-mentioned graph embedding models often only take into account the topological information of nodes but do not well incorporate the characteristic information of nodes themselves. The GAE can achieve the best predictions, mainly because it uses the graph convolution neural network to learn the characteristics of nodes in an end-to-end way. At the same time, the GAE has better robustness and stability, together with good learning effect for poor datasets.

### Comparison of the Performance of Various Classifiers

**FIGURE 6:**
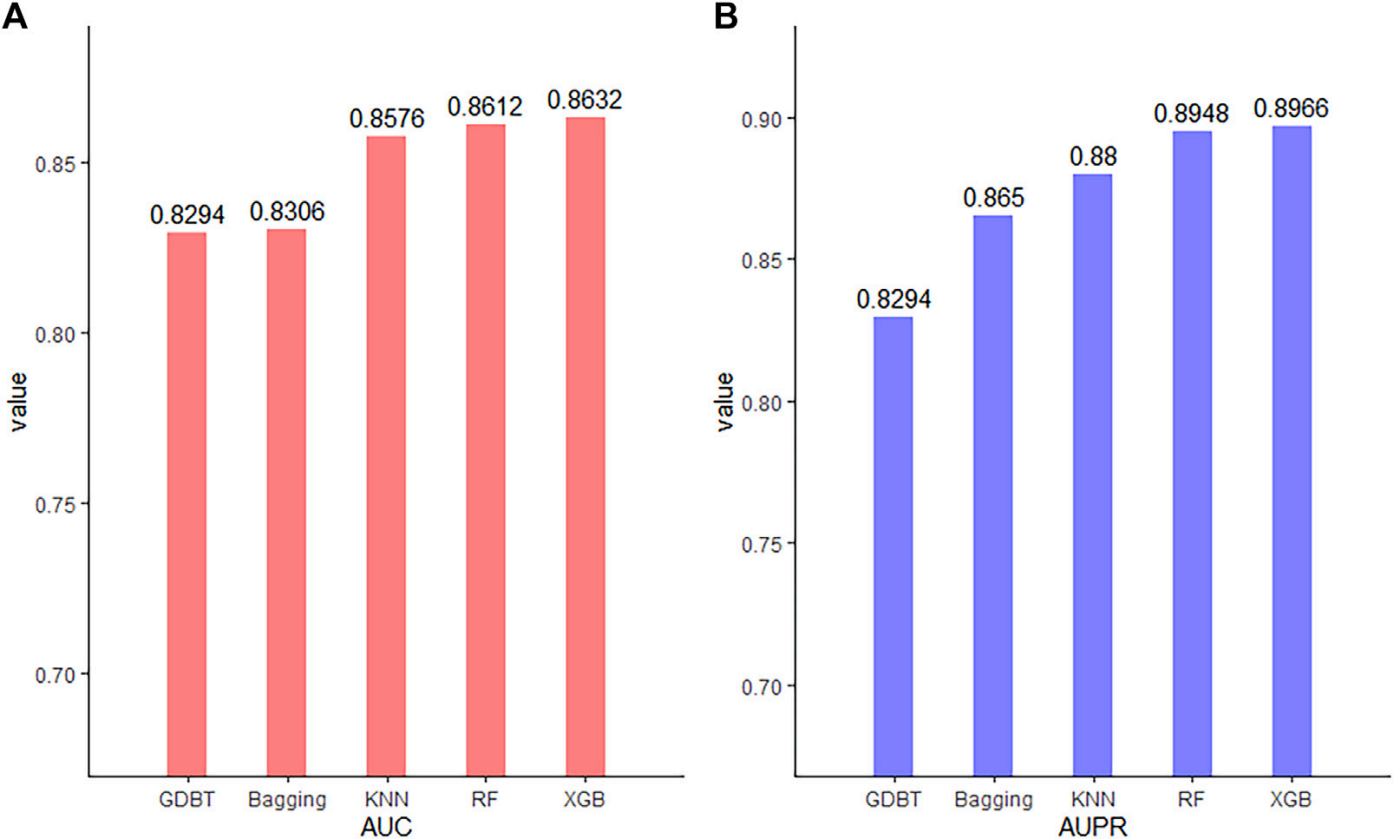
AUC **(A)** and AUPR **(B)** using various classifiers.

Classifiers play a key role in the model. To compare the prediction performance of our model under different classifiers and select the best classifier, we seek to check its predictive performances with five representative classifiers: eXtreme Gradient Boosting (XGBoost), random forest (RF), K-nearest neighbor (KNN), bagging, and gradient boosting decision tree (GBDT). The AUC and AUPR were used to evaluate their performance. As shown in Figure 6, while all the classifiers have an AUC and AUPR above 0.8, XGBoost yields the best performance. Thus, XGBoost is most suitable for our model.

### Comparison of GAE With Various Setups of Hidden Units

**FIGURE 7:**
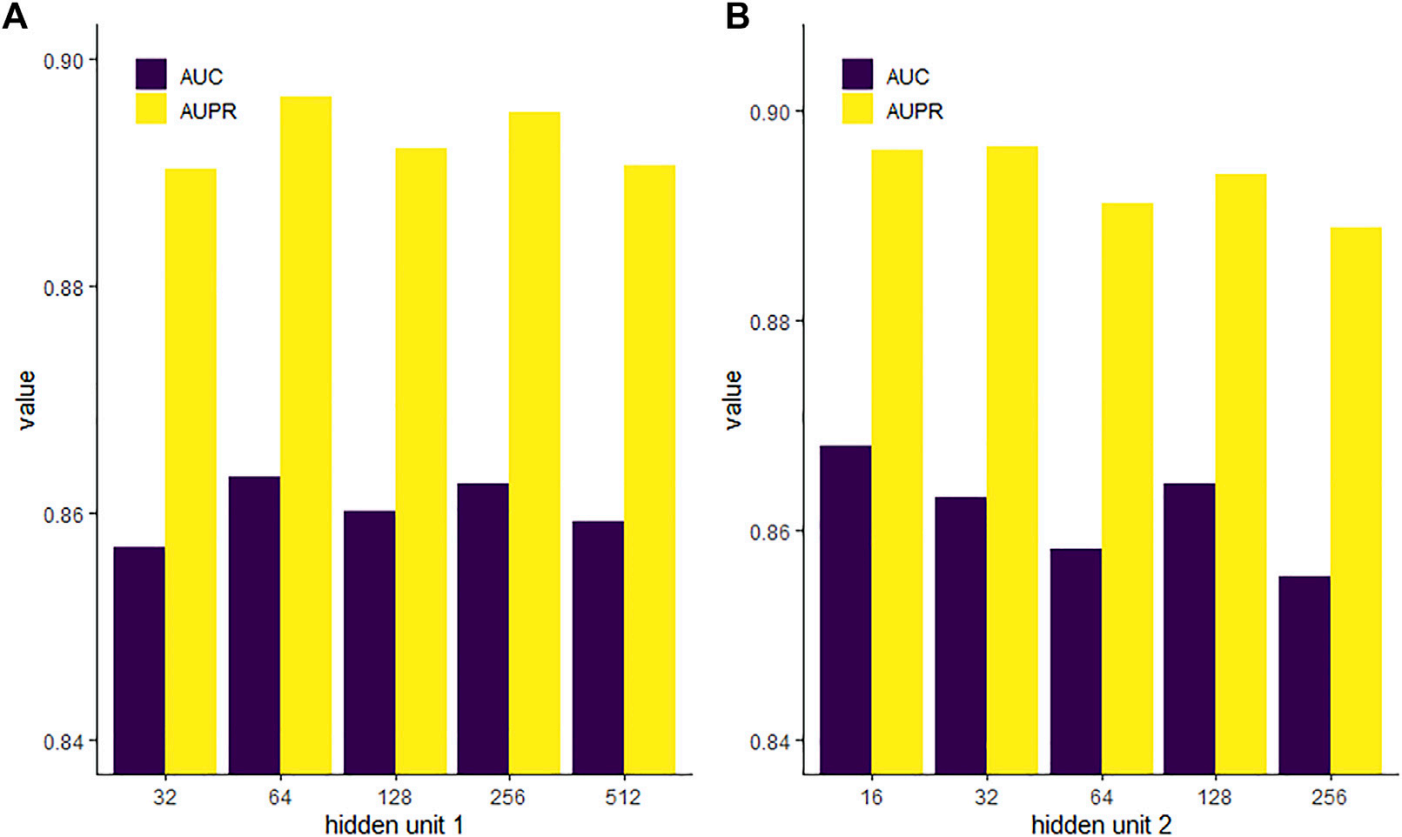
AUC and AUPR of various hidden unit setups in the first **(A)** and second **(B)** layers of GAE.

The GAE contains two layers of hidden units in the neural network. We evaluated the impact of different dimensions of each layer on the performance of the model. We fixed the second hidden layer with 32 units and then set the first hidden layer with units of 32, 64, 128, 256, and 512, respectively. Figure 7 shows that when the first hidden unit is 64, the GAE has the best performance. Then, we set the first hidden layer with units of 64 and set the second hidden layer with units of 16, 32, 64, 128, and 256, respectively. We found that model performance was slightly improved with the decrease of the unit number. The AUPR is highest when the unit number is reduced to 32, and the AUC is highest when the unit number is reduced to 16. High-dimensional representation may lead to data sparsity, which is not conducive to classification. While reducing dimension can improve the training speed of the model, dimensions too low may cause loss of key information. For the task of PMA prediction, we chose the first hidden unit to be 64 and the second hidden unit to be 32.

### Effect of Ratio of Positive to Negative Samples

Unbalanced test sets containing too many negative samples may affect the performance of the model. To explore the impact of this data imbalance on PMGAE, we used various setups of positive: negative sample ratios. In the five-fold cross validation, we constructed 1:1, 1:2, 1:5, 1:10, and 1:20 test sets by changing sizes of potentially negative samples. Table 2 shows the experimental results. The test set with different proportions has a moderate effect on the results. It suggests that, for the evaluation of model performance in predicting PMAs, the influence of different positive: negative sample ratios cannot be omitted.

**TABLE 2:** Model performance under various setups of positive: negative sample ratios.

Evaluation metrics	Positive: negative sample ratio				
	1:1	1:2	1:5	1:10	1:20
AUC	0.8632	0.8548	0.8557	0.8596	0.8626
AUPR	0.8966	0.8388	0.7653	0.7193	0.6693
Acc.	0.8015	0.8523	0.9218	0.9554	0.9753
Sen.	0.6592	0.6008	0.5594	0.5419	0.5196
Spec.	0.9437	0.9782	0.9943	0.9968	0.9981
Prec.	0.9216	0.9323	0.9513	0.9447	0.9323
MCC	0.6292	0.6646	0.6938	0.6965	0.6858

### Case Studies

Exploring cases of PMAs is of great significance to provide insights for research of diseases. Seeking support of our predictions from independent sources can evaluate the effectivity and robustness of PMGAE. For the case study, we used all other associations that did not contain three pseudogenes RPLP0P2, HLA-H, and HLA-J to train the model and then predicted the probability of all miRNAs associated with each of these three pseudogenes. The top 15 predicted associations were used to verify the predictions through starBase.

Three pseudogenes, RPLP0P2, HLA-H, and HLA-J, were used for case studies. RPLP0P2 is a pseudogene associated with a variety of cancers including lung adenocarcinoma and colorectal cancer. Several studies have shown that low expression of RPLP0P2 can lead to decreased proliferation and adhesion of tumor cells (Chen et al., 2016; Yuan et al., 2021). Table 3 shows the top 15 candidate miRNAs associated with RPLP0P2, 11 of which are supported by starBase.

**TABLE 3:** The top 15 candidate miRNAs associated with pseudogenes RPLP0P2, HLA-H, and HLA-J and the evidence from starBase.

Rank	RPLP0P2		HLA-H		HLA-J	
	miRNA	starBase	miRNA	starBase	miRNA	starBase
1	hsa-miR-15a-5p	Yes	hsa-miR-15a-5p	Yes	hsa-miR-497-5p	Yes
2	hsa-miR-424-5p	Yes	hsa-miR-15b-5p	Yes	hsa-miR-424-5p	Yes
3	hsa-miR-15b-5p	Yes	hsa-miR-16-5p	Yes	hsa-miR-195-5p	Yes
4	hsa-miR-195-5p	Yes	hsa-miR-195-5p	Yes	hsa-miR-16-5p	Yes
5	hsa-miR-497-5p	Yes	hsa-miR-497-5p	Yes	hsa-miR-15b-5p	Yes
6	hsa-miR-16-5p	Yes	hsa-miR-424-5p	Yes	hsa-miR-15a-5p	Yes
7	hsa-miR-34c-5p	No	hsa-miR-199b-5p	No	hsa-miR-23c	Yes
8	hsa-miR-449a	No	hsa-miR-3619-5p	Yes	hsa-miR-103a-3p	No
9	hsa-miR-378b	No	hsa-miR-761	Yes	hsa-miR-204-5p	No
10	hsa-miR-320c	Yes	hsa-miR-106b-5p	No	hsa-miR-3619-5p	Yes
11	hsa-miR-761	Yes	hsa-miR-125a-5p	Yes	hsa-miR-134-5p	Yes
12	hsa-miR-99a-5p	No	hsa-miR-4319	Yes	hsa-miR-613	No
13	hsa-miR-320d	Yes	hsa-miR-146a-5p	No	hsa-miR-29b-3p	Yes
14	hsa-let-7d-5p	Yes	hsa-miR-875-5p	Yes	hsa-miR-125b-3p	No
15	hsa-let-7b-5p	Yes	hsa-miR-503-5p	Yes	hsa-miR-761	Yes

HLA-H is a kind of transmembrane molecule, and it can mobilize HLA-E at the cell surface of multiple immune cells (Jordier et al., 2019). At the same time, HLA-H gene mutations cause many cases of hereditary hemochromatosis. Table 3 shows the top 15 candidate miRNAs associated with HLA-H, 12 of which are proved by starBase.

HLA-J is also a class of HLA gene. HLA-J has an immunosuppressive effect and is potentially a predictor of breast cancer (Würfel et al., 2020). Besides, HLA-A has been shown to be associated with schizophrenia. The presence of HLA-AM80468 significantly reduces the incidence of schizophrenia, whereas the presence of HLA-JM80469 increases the incidence of schizophrenia (Gu et al., 2013). As shown in Table 3, 11 of the top 15 candidate miRNAs associated with HLA-J are proved by starBase.

## Discussion

Genome-wide prediction of PMAs has great significance in both biology and medicine. It can not only help us understand the cellular role of pseudogenes but also provide clues and directions for the clinical treatment of various diseases. In this work, full potential PMAs are predicted for the first time. Feature fusion and GAE were used to construct the model, PMGAE. The performance of PMGAE was evaluated by five-fold cross validation, with an AUC of 0.8634 and AUPR of 0.8966 obtained. Extensive experiments on feature fusion, model framework, and setup were conducted.

The good performance of PMGAE may be attributed to the optimization of each step and flexibility together with the good interpretability of the model. First, we integrated the attribute information from different perspectives of nodes by feature fusion. Subsequently, the GAE was used to integrate the correlation information and attribute information to obtain the low-dimensional representation of nodes. Finally, we selected the most suitable classifier for the model as an association prediction task. By comparative experiments on the feature construction, embedding method, and classifiers, the best integrated model can be selected. The resultant PMGAE model has the optimal effect in predicting the PMAs.

In the ceRNA network, pseudogene–miRNA is the only pair of relationships that have not been studied computationally. By predicting PMAs for the first time, using PMGAE, our work fills the gap in the ceRNA network, so that all known relational pairs in the ceRNA network can be predicted by computational methods. The completed map will facilitate the studies of ceRNA network architecture and its biological implications.

Based on the successful application of PMGAE, there is space for further improvement. First, only one type of feature for each node was used when constructing a similarity feature profile. Fusing more types of node features may provide more information for model training. Second, one can also introduce intermediate layers to incorporate pseudogene–lncRNA associations and lncRNA–miRNA associations. Whether adding intermediate layers will improve the prediction effect of the model is a problem worth further exploration. Third, when constructing negative samples, we simply used non-positive samples as potential negative samples and then randomly extracted them. How to build negative samples more accurately is also a question worth exploring. Fourth and more importantly, in PMGAE, embedding and classifier are sequentially, also separately trained. For the task of PMA prediction, end-to-end modeling seeking a global optimal solution is worth further exploration. Toward a full description and understanding, we will incorporate all relation pairs to build a complete graph of the ceRNA network, together with diverse information of all types of nodes.

## Data Availability

The original contributions presented in the study are included in the article/Supplementary Material, and further inquiries can be directed to the corresponding author.
